# A Comparison of GFR Calculated by Cockcroft-Gault vs. MDRD Formula in the Prognostic Assessment of Patients with Acute Pulmonary Embolism

**DOI:** 10.1155/2021/6655958

**Published:** 2021-12-08

**Authors:** Magdalena Pływaczewska, David Jiménez, Mareike Lankeit, Piotr Pruszczyk, Maciej Kostrubiec

**Affiliations:** ^1^Department of Internal Medicine and Cardiology, Centre for Management of Venous Thromboembolic Disease, Medical University of Warsaw, Warsaw, Poland; ^2^Respiratory Department and Medicine Department, Ramón y Cajal Hospital, IRYCIS and Alcalá de Henares University, Madrid, Spain; ^3^CIBER Enfermedades Respiratorias (CIBERES), Madrid, Spain; ^4^Clinic of Cardiology and Pneumology, University Medical Center Göttingen, Germany; ^5^Department of Internal Medicine and Cardiology, Campus Virchow Klinikum (CVK), Charité-University Medicine Berlin, Berlin, Germany; ^6^Center for Thrombosis and Hemostasis, Johannes Gutenberg University of Mainz, Mainz, Germany

## Abstract

**Introduction:**

Risk stratification is mandatory for optimal management of patients with acute pulmonary embolism (APE). Previous studies indicated that renal dysfunction predicts outcome and can improve risk assessment in APE.

**Aim:**

The aim of the study was a comparison of estimated glomerular filtration rate (eGFR) formulas, MDRD, and Cockcroft-Gault (CG), in the prognostic assessment of patients with APE.

**Materials and Methods:**

Data from 2274 (1147 M/1127 F, median 71 years) hospitalised patients with APE prospectively included in a multicenter, observational, cohort study were analysed. A serum creatinine measurement as a routine laboratory parameter at the cooperating centers and eGFR calculation were performed on admission. Patients were followed for 180 days. The primary outcome was death from any cause within 30 days.

**Results:**

The eGFR levels assessed by both, MDRD (eGFR_MDRD_) and CG formula (eGFR_CG_), were highest in patients with low-risk APE and lowest in high-risk APE. The eGFR (using both methods) was significantly lower in nonsurvivors compared to survivors. Using a threshold of <60 ml/min/1.73 m^2^, eGFR_MDRD_ revealed the primary outcome with sensitivity 67%, specificity 52%, PPV 8%, and NPV 97%, while eGFR_CG_ had a sensitivity 62%, specificity 62%, PPV 8.6%, and NPV 96%. The area under the ROC curve for eGFR_CG_ tended to be higher than that for eGFR_MDRD_: 0.658 (95% CI: 0.608-0.709) vs. 0.631 (95% CI: 0.578-0.683), *p* = 0.12. A subanalysis of ROC curves in a population above 65 yrs showed a higher AUC for eGFR_CG_ than based on MDRD. Kaplan-Meier analysis showed a worse long-term outcome in patients with impaired renal function.

**Conclusion:**

eGFR_MDRD_ and eGFR_CG_ assessed on admission significant short- and long-term mortality predictors in patients with APE. The eGFR_CG_ seems to be a slightly better 30-day mortality predictor than eGFR_MDRD_ in the elderly.

## 1. Introduction

Risk stratification is mandatory for the optimal management of patients with acute pulmonary embolism (APE). While the treatment of patients with high-risk APE according to the European Society of Cardiology (ESC) guidelines [1] is apparent and acknowledged, normotensive patients constitute an extensive category of low to moderate risk with challenging and disputed management. Non-high-risk patients are further classified based on plasma biomarkers and imaging studies into intermediate-high- and intermediate-low-risk patients, but their prognosis is still ambiguous. The previous observation showed that the mortality rate did not differ significantly between patients in these two groups [2].

APE affects not only pulmonary circulation but also systemic circulation and the functions of other organs due to hypoxemia and increased venous pressure. Kidneys are one of the most sensitive body organs for hypoxemia [3]. Estimated glomerular filtration rate (eGFR) is a risk factor of mortality in cardiovascular disease [4–6]. Further, renal dysfunction has been shown to be a prognostic marker in APE [7]. Previous studies indicated that renal dysfunction predicts short- and long-term outcomes and can improve the risk assessment in APE [8, 9].

A prime parameter used to assess renal function is the glomerular filtration rate (GFR) based on a kidney excretion of serum creatinine. The MDRD (Modification of Diet in Renal Disease) [10] is one of the most often used formula for GFR estimation and determination of renal insufficiency; nevertheless, the Cockcroft-Gault (CG) formula [11] was suggested to be more precise in elderly patients with low body weight [12]. Of note, following the common practice in the majority of clinical trials, the CG formula is recommended for tapering a medication dose for the sake of impaired renal function. The aim of the study was a comparison of two GFR estimation formulas (MDRD and CG formulas) in terms of prognostic assessment.

## 2. Materials and Methods

The multicenter, observational, cohort study included patients hospitalised with APE at three cooperating European centres in three countries (see Acknowledgements section). Diagnosis of APE was established based on the high clinical probability of APE, contrast-enhanced computed tomography (CT), or ventilation/perfusion lung scan, presence of thrombi in the right atrium, ventricle or proximal pulmonary artery in echocardiography, or presence of proximal deep vein thrombosis on ultrasound in patients with APE symptoms. Patients underwent clinical assessment of hemodynamic status and presence of hypoxia (defined as arterial oxyhemoglobin saturation < 95% measured on admission or necessity of oxygen supplementation). The simplified Pulmonary Embolism Severity Index (sPESI) was calculated for all patients [13]. Missing data were considered to be normal. We classified patients into either low-risk (0 points) or intermediate-risk of death (≥1 points) groups, to compare mortality in those groups after adding the eGFR threshold. Next step was the assessment of the short-term mortality risk according to ESC guidelines [1]. Patients' data were published in the previous manuscript [9].

Venous plasma and serum samples were collected on admission. Creatinine measurement was performed as a routine laboratory parameter at the Departments of Clinical Chemistry of the University of Göttingen, the Central Laboratory of Infant Jesus Teaching Hospital, Warsaw, Poland, and the Biochemistry Department at Ramón y Cajal Hospital, Madrid, Spain.

GFR was estimated by the MDRD equation [10]: eGFR_MDRD_ = 175 × (standardized serum creatinine)^−1.154^ × (age in years)^−0.203^ × 0.742 [if female] × 1.212 [if Black], and the CG formula [11]: eGFR_CG_ = (140 − age in years) × (weight in kilograms) × (0.85 if female)/(72 × serum creatinine).

Cardiac troponin I elevation was defined as the value of above the 99^th^ percentile of healthy subjects with a coefficient variation of 10% for the used test. Elevation of N-terminal probrain natriuretic peptide (NT-pro-BNP) was defined according to local laboratory standards.

Transthoracic echocardiogram (TTE) was performed within 48 hours of PE diagnosis. The presence of right ventricle (RV) dysfunction was defined as dilatation of the right ventricle in relation to left ventricle (LV) (RV/LV ratio > 1.0 from the subcostal or apical four-chamber view) coexisting with the absence of the inspiratory collapse of the inferior vena cava or an elevated maximal systolic gradient through the tricuspid valve (>30 mmHg), without significant left ventricular dysfunction [14].

Decisions on the therapy remained at the discretion of the managing physicians. Initial anticoagulant treatment included the following: intravenous unfractionated heparin (UFH), subcutaneous body weight-adjusted dose of low-molecular-weight heparin (LMWH), or non-vitamin K-dependent oral anticoagulant (NOAC). Thrombolytic, interventional, or surgical treatment was administered when it was necessary, according to the team in charge.

All patients included in the study were followed for 180 days. The primary outcome of the study was defined as death from any cause within 30 days after the admission. The secondary outcomes were 180-day all-cause mortality and major and clinically relevant nonmajor bleeding. Clinically relevant bleeding was defined based on the criteria of the International Society of Thrombosis and Hemostasis (ISTH) [15, 16]. Data after six months was obtained by clinical examination during follow-up visits or by a telephone conversation with the patient or his/her treating physician.

The cause of death was adjudicated by three of the authors (M.L., D.J., and M.K.) by reviewing patients' medical records and the results of an autopsy if performed. Death was determined to be PE-related if it was confirmed by autopsy or if it followed a clinically severe PE episode, either immediately or shortly after an objectively confirmed recurrent event and in the absence of an alternative diagnosis.

### 2.1. Statistical Analyses

Data characterised by a normal distribution are expressed as mean followed by standard deviation, while data with abnormal distribution are expressed by median with quartiles from first to third [Q1-Q3]. Student's or Mann–Whitney's tests were used for comparisons between two groups, while comparisons between more than two groups were performed by ANOVA or Kruskal–Wallis tests. Fisher's or chi-squared tests were used to compare discrete variables, as appropriate.

Receiver Operating Characteristic (ROC) analysis was used to determine the area under the curve (AUC) of GFR estimated by MDRD and CG formulas with regard to the study outcomes. Sensitivity, specificity, positive and negative predictive values, and the positive and negative likelihood ratios for different eGFR cut-off values and dichotomous/dichotomised variables were calculated and presented with 95% confidence intervals (CIs). The cut-off value for eGFR was chosen a prior as <60 ml/min/1.73 m^2^ according to the Kidney Disease Outcomes Quality Initiative (KDOQI) classification [17] and according to the previous study conducted in one of the centres [9].

Kaplan–Meier analysis was used to investigate cumulative 30-day and 180-day survival rates with analysis of statistical signification for eGFR chosen cut-off points. The impact of eGFR and other clinical factors on mortality were evaluated using univariable Cox proportional-hazard regression. Forward stepwise selection with a 0.1 level for staying in the model was used to identify significant predictors in multivariable analysis.

Data were considered significant at *p* < 0.05. Statistica (StatSoft 13.3, Inc. 2016, Tulsa, OK, USA), MedCalc, and PQStat software were used for statistical calculations.

### 2.2. Ethics Approval

The Bioethics Committee at the Medical University of Warsaw has given approval to conduct the study.

## 3. Results

### 3.1. General Characteristics

To the study were included 2274 (1147 M/1127 F, median 71 years, range: 18-101) patients.  Table 1 presents the clinical characteristics of patients. Transthoracic echocardiogram was performed in 1436 (63%) patients; of these, 768 (53%) were diagnosed with RV dysfunction. Thrombolysis was administered to 147 (6%) patients, resuscitation was performed in 33 (1.5%), while 117 (5%) needed catecholamine infusion during the hospitalisation. One hundred twenty-eight patients (5.6%) died during the 30-day follow-up, including 69 (3%) patients whose cause of death was found PE-related. Recurrent PE occurred in 28 (1.2%) patients during the 30-day follow-up. In Table 1, we compared clinical parameters between two thresholds (<60 and ≤30) with the same GFR estimation methods to show that patients with lower eGFR calculated with the same method more often have comorbidities or elevated levels of biomarkers.

### 3.2. Glomerular Filtration Rate and Mortality

The GFR levels assessed by both MDRD (eGFR_MDRD_) and CG formula (eGFR_MDRD_) were highest in patients with low-risk APE and lowest in patients with high-risk APE. In patients with low risk APE, eGFR_MDRD_ was 80 (4-192) ml/min/1.73 m^2^ vs. 85 (6-247) ml/min calculated by CG formula (*p* < 0.001); in intermediate-risk APE, 71 (4-297) ml/min/1.73 m^2^ vs. 66 (6-258) ml/min, *p* < 0.001, and 58 (13-157) ml/min/1.73 m^2^ vs. 54 (13-220) ml/min, *p* = 0.86, in high-risk APE, respectively.

**Figure 1:**
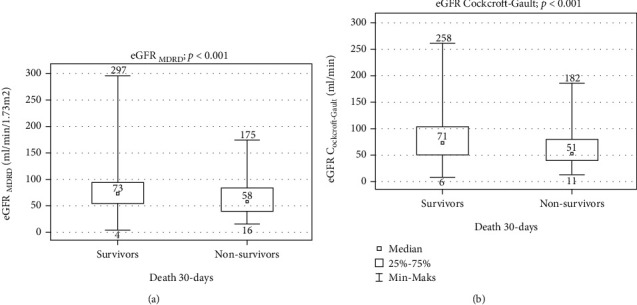
(a) The comparison of calculated GFR MDRD in nonsurvivors to survivors. (b) The comparison of calculated GFR CG in nonsurvivors to survivors.

The calculated eGFR (eGFR_MDRD_ and eGFR_CG_) was significantly lower in nonsurvivors compared to survivors, 55 (16-175) vs. 73 (4-297) ml/min/1.73 m^2^, *p* < 0.001, for eGFR_MDRD_ and 51 (11-182) vs. 71 (6-258) ml/min, *p* < 0.001, for GFR_CG_ (Figures 1(a) and 1(b)).

**Figure 2:**
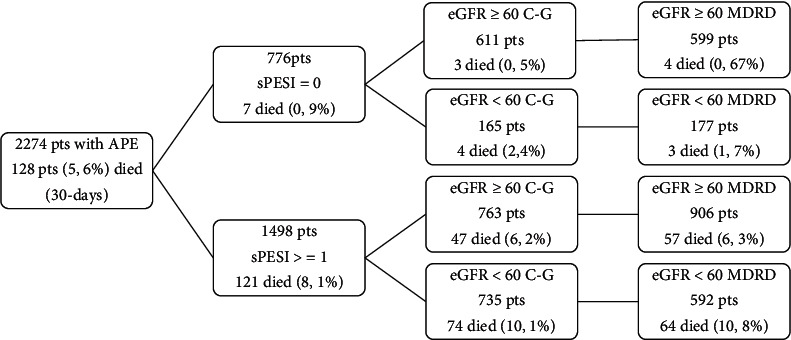
The 30-day mortality according to the kidney function and sPESI score.

Figure 2 illustrates the 30-day mortality rates of patients according to the kidney function. In patients with intermediate-low and intermediate-high risk according to the risk stratification algorithm proposed by the ESC guidelines, mortality rates were similar.

**Figure 3:**
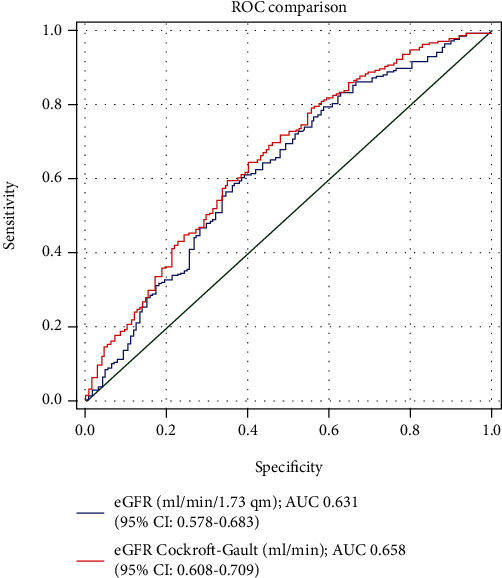
A comparison of the area under the ROC curve (AUC) for eGFR_CG_ and for eGFR_MDRD_.

A comparison of the area under the ROC curve (AUC) for eGFR_CG_ and for eGFR_MDRD_: 0.658 (95% CI: 0.608 to 0.709) vs. 0.631 (95% CI: 0.578 to 0.683) showed marginal difference (*p* = 0.12) (Figure 3). Two thresholds, eGFR < 60 ml/min or ml/min/1.73 m^2^ and eGFR ≤ 30 ml/min or ml/min/1.73 m^2^, were considered in subsequent analyses.

The additional evaluation of kidney function allowed a more comprehensive risk assessment regardless of the GFR calculation method. Moreover, both tests improved ESC classification based on sPESI score and reclassified patients with almost the same accuracy; the net reclassification index (NRI) for sPESI score with eGFR_MDRD_ < 60 ml/min/1.73 m^2^ vs. sPESI with eGFR_CG_ < 60 ml/min was 0.03. The eGFR threshold of <60 ml/min/1.73 m^2^ revealed the primary outcome with sensitivity 67%, specificity 52%, PPV 8%, and NPV 97% if calculated by MDRD while a eGFR_CG_ < 60 ml/min was associated with a sensitivity 62%, specificity 62%, PPV 8.6%, and NPV 96%.

### 3.3. Short-Term Regression Analysis

Univariate analysis of Cox proportional-hazard regression for eGFR_MDRD_ < 60 ml/min/1.73 m^2^ showed HR 1.73 (95% CI: 1.33 to 2.27, *p* < 0.001) vs. 2.08 (95% CI: 1.58 to 2.73, *p* < 0.001) by CG in prediction of the primary outcome. Of note, for eGFR_MDRD_ ≤ 30 ml/min/1.73 m^2^, HR was 1.46 (95% CI:0.95 to 2.23, *p* = 0.08) vs. 1.11 (95% CI: 0.8 to 1.52, *p* = 0.5) for eGFR_CG_. However, the multivariate analysis model showed statistical significance only of eGFR_CG_ < 60 ml/min, and the other remarkable risk factors were heart rate 30-day bleeding and cancer (Table 2)

In the group of 900 patients with eGFR_CG_ < 60 ml/min, 275 had eGFR_MDRD_ ≥ 60 ml/min/1.73 m^2^ and 17 (6%) of them died during the 30-day follow-up. To compere, 144 patients had eGFR_CG_ ≥ 60 ml/min and eGFR_MDRD_ < 60 ml/min/1.73 m^2^; 6 (4%) patients died in this group. Interestingly, 72 patients with eGFR_CG_ ≤ 30 ml/min had eGFR_MDRD_ > 30 ml/min/1.73 m^2^, and 12 (16%) of them died within 30 days of follow-up. On the other hand, in 34 patients having eGFR_MDRD_ ≤ 30 ml/min/1.73 m^2^, it was above 30 according to Cockcroft-Gault, but only 1 (2%) patient died in this group for 30 days. For both cut-off points (eGFR ≤ 30 and eGFR < 60), patients with lower eGFR_CG_ and higher eGFR_MDRD_ were older and had higher sPESI score, D-dimer level, troponin, and NT-proBNP concentration.

### 3.4. Long-Term Outcome

**Figure 4:**
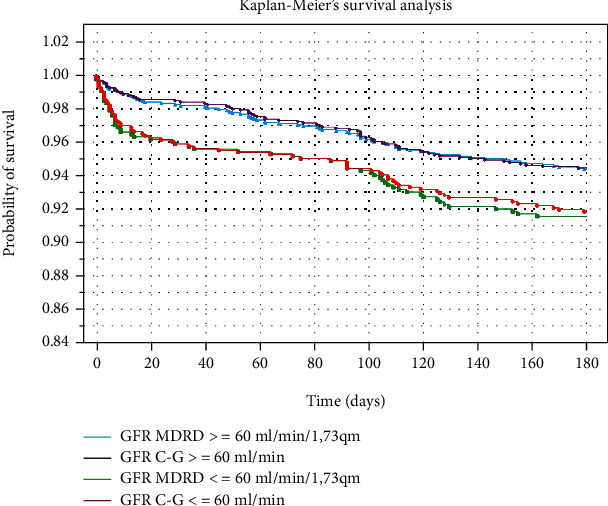
Kaplan-Maier analyses of 180-day survival for proper and impaired renal function calculated using both methods.

Kaplan-Maier curve analyses demonstrated that the probability of 30- and 180-day survival was lower for patients with impaired renal function calculated by both methods (Figure 4). In multivariate analysis eGFR_CG_ ≤ 30 ml/min, cancer and the elevated troponin I level were shown to be the long-term predictors of the 180-day mortality (Table 2(b)).

### 3.5. Elderly Group

Patients over 65 years old constituted 1428 (63%) of the study group. The median age for the group was 77 within the range 65 to 101. The gender ratio was equal (714 M/714 F).

The subanalysis of ROC curves for the population above 65 showed higher AUC for eGFR_CG_ than eGFR_MDRD_, 0.673 (95% CI:0.615-0.731) vs. 0.631 (95% CI:0.569-0.692), *p* = 0.04, respectively.

### 3.6. Bleeding

During the 30-day follow-up, we observed 135 bleeding episodes.  Table 3 shows the incidence of bleedings (clinical relevant bleeding nonmajor and major bleeding) with regard to eGFR. Impaired renal function was a significant risk factor for the occurrence of bleeding in the short-term observation and haemorrhage occurred approximately three times higher in patients with eGFR ≤ 30 ml/min or ml/min/1.73 m^2^ calculated by both methods in comparison to other patients. However, the usage of different methods for calculation of eGFR did not facilitate the distinction of a group with more frequent bleeding complications.

## 4. Discussion

Impaired renal function is widely known as a mortality risk factor in cardiovascular diseases.

Acute kidney injury (AKI) in patients with exacerbation of heart failure and the acute coronary syndrome is associated with worse short- and long-term prognoses [4, 5]. Therefore, serum plasma creatinine is included into the GRACE Score [6]. In patients with APE, kidney dysfunction also remains a marker of poor prognosis. In the ICOPER, creatinine > 177 *μ*mol/l was the 3-month mortality predictor [18]. The Hestia Study investigators proved that patients with APE and estimated creatinine clearance by CG < 30 ml/min should be hospitalised due to increased risk [19]. The previous study conducted in our department revealed that the inclusion of eGFR improved troponin-based prediction of early mortality in APE patients [8]. Moreover, the inclusion of eGFR ≤ 60 ml/min/1.73 m^2^ enhanced the ESC risk stratification model with NRI of 0.42 [9].

The current observational, multicenter cohort study confirmed that eGFRs is an indicator of worse outcome in patients with APE. The eGFR cut-off point was predefined; nevertheless, the analysis of the present ROC curves also suggested a cut-off point close to the chosen hitherto. The eGFRs < 60 ml/min/1.73 m^2^ or ml/min calculated by both methods, MDRD and Cockcroft-Gault, were significant predictors of 30-day mortality and revealed the primary outcome with very high NPV of 96% but with low PPV, approximately 9%. The area under the ROC curve for eGFR calculated with the Cockcroft-Gault formula was similar to GFR estimated with MDRD. Still, the subanalysis suggests that the CG-based eGFR could be superior to eGFR by MDRD in the elderly population. Overall, we can conclude that eGFR is a congruent predictor regardless of estimation method.

Neither serum creatinine nor calculated eGFR are relevant to diagnose early kidney injury; also, they do not enable distinguishing acute from chronic renal failure [20]. Additionally, an increase of serum creatinine primarily reflects somewhat the functional changes in glomerular filtration and is not an accurate injury marker. Patients with acute heart failure and worsening renal function (WRF) without clinical implication had a similar outcome as those with normal renal function [21]. In response to these reasons, novel, more specific markers of impaired renal function have been proposed.

The recent publication showed that urinary neutrophil gelatinase-associated lipocalin (uNGAL) and kidney injury molecule-1 (uKIM-1) are makers of true-WRF and worse outcome in patients with acute heart failure [22]. Similarly, serum levels of N-GAL and cystatin C are mortality predictors in patients with APE [7]. The novel markers regardless of being more specific and accurate to detect renal insufficiency are also expensive and not widely available. Thus, creatinine and eGFR will probably remain a rational choice for renal function assessment.

The KDIGO recommended eGFR estimating equations above SCr alone because it provides a more direct assessment of glomerular filtration [20]. In previous studies, we used eGFR calculated with the MDRD equation [7–9] as the most popular for the evaluation of renal function. The MDRD equation underestimated the eGFR and is not precise enough in a higher range of eGFR [23]. On the other hand, the CG formula cannot be accurate enough for a low value of eGFR [24, 25]. Additionally, eGFR_CG_ relies on total body weight and so overestimates eGFR in overweight or obese patients [26, 27].

However, Michels et al. showed in the group of 271 patients that eGFR calculated by CG formula had higher accuracy than MDRD or CKD-EPI for elderly patients with lower body mass [28]. In our study, the median age was 71 years and the median BMI was 26 kg/m^2^; it failed to prove the superiority of the CG-based GFR in the APE risk stratification.

The discrepancy of the diagnosis of severe renal impairment between the CG and CKD-EPI formulas was also noticed in a recent analysis of 41,796 patients from the RIETE registry. Among the 4676 patients with eGFR ≤ 30 ml/min (or ml/min/1.73 m^2^) according to at least one of the formulas, this was not confirmed by the other formula in 1904, leading to discordant results in 40.7% [29]. Nevertheless, irrespective of the used formula, patients with severe renal impairment had a higher rate of major bleedings being anticoagulated (approximately 10% vs. 4%). The all-cause mortality rates were remarkably higher in subgroups with low eGFR than in patients without severe renal impairment according to both formulas, but the all-cause mortality rate was also significantly higher in comparison to the CG+CKD-EPI subgroup with the CKD-EPI-only subgroup (24% vs. 13%).

Patients with APE classified to intermediate-risk class are the most heterogeneous group, although the previous attempt to divide this broad category into two subgroups, intermediate-high and intermediate-low risk [2], was unsuccessful to distinguish a group of higher mortality. Altinsoy et al. [30] presented in the multivariate analysis that GFR estimated by CKD-EPI or MDRD together with positive troponin concentration are independent predictors of adverse outcome in normotensive patients with APE. Moreover, eGFR was correlated with RV dysfunction. However, our results suggest that the addition of eGFR with excellent NPV to the assessment model improved the risk stratification, especially indicating patients with the probable good outcome.

### 4.1. Study Limitations

The presented cohort includes patients with comorbidities, also with known chronic kidney disease. Moreover, contrast-enhanced CT scanning was performed to diagnose PE in the majority of patients. To avoid the potential influence of contrast on the assessment of renal function, blood samples drawn on the admission or shortly after scanning were used for creatinine assay and GFR calculation.

The Cockcroft-Gault equation has not been expressed using standardized creatinine values.

We have included patients with a surgical treatment of APE (15 patients, 0.6%), interventional thrombus fragmentation (6 patients, 0.2%), and thrombolysis (147, 6%). Despite the fact of a small number of invasive-treated patients, this may affect the results for the entire population. The aim of the study was to present the general population of patients with APE without restrictive exclusion criteria.

The performed analysis did not compare the groups of patients depending on the selected anticoagulant treatment. Among the entire population, over 50% (1181) of patients were initially treated with low molecular weight heparin and at least 119 patients with unfractionated heparin. The data on this subject are incomplete. The database does not contain complete data on the final choice of anticoagulant therapy, including selected NOACs.

## 5. Conclusions

The GFR assessed on admission in patients with APE by both MDRD and Cockcroft-Gault formulas is a significant short- and long-term mortality predictor. The GFR estimated by the Cockcroft-Gault formula seems to be a potentially better 30-day mortality predictor than GFR calculated by MDRD in the elderly.

## Figures and Tables

**Table 1:** General characteristic of all patients and depending on glomerular filtration rate.

	All pts. *N* = 2274	eGFR_MDRD_ < 60 (ml/min/1.73 m^2^) *N* = 769	eGFR_MDRD_ ≤ 30 (ml/min/1.73 m^2^) *N* = 106	*p* value	eGFR_CG_ < 60 (ml/min) *N* = 900	eGFR_CG_ ≤ 30 (ml/min) *N* = 144	*p* value
*Characteristics*							
Age (years)	71 (18-101)	76 (20-101)	77 (20-94)	0.004	79 (20-101)	83 (20-101)	0.0001
Sex (male/female)	1148 (50.4%)/1126 (49.6%)	238 (31%)/531 (69%)	38 (36%)/68 (64%)	0.002	385 (43%)/515 (57%)	56 (39%)/88 (61%)	0.004
BMI (kg/m^2^) (*n* = 2013)	26.6 [23.8-30.1]	26.6 [24.2-30.1]	26.9 [23.9-30.1]	Non-significant (NS)	25.3 [23.2-28.3]	24.2 [22.5-27.7]	0.01
HR (*n* = 2251)	90 [78-105]	88 [76-105]	93 [75-110]	NS	89 [76-103]	92 [76-110]	0.08
SBP (*n* = 2244)	130 [115-143]	130 [110-145]	115 [93-140]	0.04	130 [110-144]	119 [100-140]	NS
Creatinine (*μ*mol/l)	86.6 [70.7-106]	120.2 [101.6-145.8]	211.3 [171.5-253.7]	<0.001	107.8 [89.3-138.8]	166.2 [141.4-221]	<0.001
eGFR_MDRD_ (ml/min/1.73 m^2^)	72.5 [53.3-93]	45.5 [35.5-53.5]	24.7 [19.5-28]	<0.001	50.6 [37.4-63.4]	29.9 [22.5-36.2]	<0.001
eGFR_CG_ (ml/min)	70.1 [48.5-99]	43.1 [33.1-55.5]	24.4 [18.9-33.4]	<0.001	43.6 [34.4-51.9]	24.6 [19.3-27.4]	<0.001
NTproBNP (pmol/l) (*n* = 744)	87.1 [15.9-350.95]	275.5 [75.2-876.6]	583.4 [155.7-2155]	<0.001	300.9 [87.3-1031.5]	642.3 [143.5-1539.3]	<0.001
CHF (*n* = 2254)	232 (10%)	131 (17%)	23 (21.6%)	<0.001	156 (17%)	36 (25%)	<0.001
Pulmonary disease (*n* = 2265)	221 (9.8%)	92 (12%)	18 (17%)	0.004	105 (11%)	18 (12.5%)	NS
Cancer (*n* = 2250)	379 (17%)	129 (16%)	21 (20%)	NS	137 (15%)	21 (14%)	NS
RV/LV > 1 on TTE (*n* = 1436)	314 (22%)	118 (15%)	17 (16%)	NS	121 (13.4%)	19 (13%)	NS
Elevated troponin (*n* = 1870)	738 (39%)	305 (39%)	55 (52%)	<0.001	326 (36%)	67 (46.5%)	0.002

*Short-term outcome*							
30-day bleeding	135 (6%)	64 (8%)	15 (14%)	0.001	70 (7.7%)	17 (12%)	0.002
30-day all-cause mortality	128 (5.6%)	67 (8.7%)	15 (14%)	<0.001	78 (8.6%)	26 (18%)	<0.001
30-day PE-related mortality	69 (3%)	41 (5.3%)	12 (11%)	<0.001	49 (5.4%)	20 (14%)	<0.001

*Long-term outcome*							
180-day mortality	208 (9%)	94 (12%)	23 (21.6%)	<0.001	107 (12%)	32 (22%)	0.005

BMI: body mass index; CG: Cockcroft-Gault; CHF: chronic heart failure; eGFR: estimated glomerular filtration rate; HR: heart rate; LV: left ventricle; MDRD: Modification of Diet in Renal Disease; NS: nonsignificant; NTproBNP: N-terminal probrain natriuretic peptide; RV: right ventricle; SBP: systolic blood pressure; TTE: transthoracic echocardiogram. Values in square parenthesis represent quartiles from first to third [Q1-Q3] for the nonparametrical parameters.

**Table:** (a) Multivariate analysis risk predictors of 30-day all-cause mortality

Parameter	Hazard ratio	95% confidence interval	*p* value
eGFR_CG_ < 60 ml/min	1.82	1.33-2.50	<0.001
Heart rate (HR)	0.98	0.97-0.99	0.003
30-day bleeding	1.59	1.11-2.29	0.01
Cancer	1.53	1.08-2.19	0.02

**Table:** (b) Multivariate analysis risk predictors of 180-day all-cause mortality

eGFR_CG_ ≤ 30 ml/min	2.37	1.44-3.9	<0.001
Systolic blood pressure	0.99	0.98-0,99	<0.001
Cancer	2.06	1.46-2.99	<0.001
Elevated troponin	2.62	1.9-3.6	<0.001

**Table 3:** Bleeding occurrence in 30 days depending on eGFR.

	Number of bleeding episodes	Percent of bleeding in the group
eGFR_CG_ > 60 & bleeding	65	4.7%
eGFR_CG_ < 60 & bleeding	70	7.7%
eGFR_CG_ ≤ 30 & bleeding	17	11.8%
eGFR_MDRD_ > 60 & bleeding	71	4.7%
eGFR_MDRD_ < 60 & bleeding	64	8.3%
eGFR_MDRD_ ≤ 30 & bleeding	15	14.2%

## Data Availability

The datasets used and analysed during the current study are available from the corresponding author on reasonable request.
